# Medical infrared thermal imaging of canine appendicular bone neoplasia

**DOI:** 10.1186/s12917-019-2180-6

**Published:** 2019-12-03

**Authors:** J. Sung, C. Loughin, D. Marino, F. Leyva, C. Dewey, S. Umbaugh, M. Lesser

**Affiliations:** 1 Department of Surgery, Long Island Veterinary Specialists, 163 South Service Road, Plainview, NY 11803 USA; 2 Department of Clinical Sciences, Cornell University College of Veterinary Medicine, 930 Campus Road, Box 33, Ithaca, NY 14853 USA; 3 Computer Vision and Image Processing Laboratory, Electrical and Computer Engineering Department, Southern Illinois University Edwardsville, Edwardsville, IL 62062 USA; 4 Biostatistics Unit, Feinstein Institute for Medical Research, Northwell Health, 350 Community Dr, Manhasset, NY 11030 USA

**Keywords:** Medical infrared thermal imaging, Canine appendicular bone cancer

## Abstract

**Background:**

Medical infrared thermal imaging (MITI) is a noninvasive imaging modality used in veterinary medicine as a screening tool for musculoskeletal and neurological disease processes. An infrared camera measures the surface body heat and produces a color map that represents the heat distribution. Local trauma or disease can impair the autonomic nervous system, which leads to changes in the local dermal microcirculation and subsequent alteration of surface body heat. Disruption of autonomic flow to the cutaneous vasculature at deeper levels can also result in asymmetric thermographic results. The purpose of this study was to evaluate surface temperature differences between limbs affected by bone neoplasia and their normal contralateral limbs.

**Results:**

A statistically significant difference in average temperature was noted between regions of interest of the two groups (paired difference: 0.53 C° ± 0.14; *P* = 0.0005). In addition, pattern recognition analysis yielded a 75–100% success rate in lesion identification.

**Conclusions:**

Significant alterations noted with average temperature and thermographic patterns indicate that MITI can document discernible changes associated with the presence of canine appendicular bone tumors. While MITI cannot be used as the sole diagnostic tool for bone cancer, it can be used as a screening modality and may be applicable in early detection of cancer.

## Background

Primary canine appendicular neoplasia is a debilitating disease process that typically affects large to giant breed dogs and cause appendicular swelling, lameness, and even pathologic fractures [1–3]. Osteosarcoma is the most common primary bone tumor, accounting for more than 85% of primary bone tumor cases and approximately 2–7% of all canine tumors [2, 4–6]. Other causes of bone lesions include, but are not limited to, chondrosarcoma, fibrosarcoma, spindle cell sarcoma, histiocytic sarcoma, synovial sarcoma, hemangiosarcoma, multiple myeloma, lymphosarcoma, metastasis, bacterial osteomyelitis, fungal osteomyelitis, bone cyst, bone infarct and osteochondroma [2, 3]. While tumor behavior is dependent on tumor type, primary appendicular neoplasia generally affects geriatric patients and has a slight disposition towards males [1–3, 7]. Thoracic limbs are more commonly affected than pelvic limbs [5, 8]. Palliative care can include analgesics, bisphosphonates, chemotherapy, radiation, or limb amputation [2, 3]. Although median survival times and metastatic rates can differ depending on the tumor type, bone neoplasia bears a poor prognosis [2, 3]. Death is commonly due to metastatic disease despite treatment options such as surgery or radiation for local disease or chemotherapy [3].

Patients with bone neoplasia typically present with subtle and nonspecific clinical signs such as appendicular swelling or mild weightbearing lameness. In severe cases, a non-weightbearing lameness due to a pathologic fracture may be noted. Three percent of all fracture cases are pathologic in origin [8, 9]. Various imaging modalities have been described to help with diagnosis as well as with staging, surgical planning, and radiation treatment [2]. Radiography is commonly accessible in most veterinary clinics to determine the nature and degree of bone pathology. Osteolysis can be suggestive of a pathology but can be difficult to detect, especially in early stages of neoplasia. It has been reported that osteolysis is radiographically evident in humans when 30–70% of the trabecular bone is destroyed [10, 11]. Computed tomography (CT) is a more sensitive modality for evaluation of osteolysis but is costly and not as readily available [10, 11]. Other advanced imaging techniques such as magnetic resonance imaging (MRI), nuclear scintigraphy, positron emission tomography (PET), and single-photon emission computed tomography (SPECT) can also be used to evaluate musculoskeletal neoplasia but are not available to most veterinarians, require general anesthesia, and are significantly more costly for owners to pursue [2]. Definitive diagnosis is based on histopathologic evaluation of bone samples obtained via bone biopsy or limb amputation [2, 3].

Medical infrared thermal imaging (MITI), also known as medical infrared imaging (MII) or infrared thermography (IRT), is a noninvasive imaging modality used in human and veterinary medicine by quantitatively detecting alterations in surface heat distribution, typically secondary to injury or other disease processes [12, 13]. Historically, MITI has been use in large animal medicine to evaluate various etiologies of lameness such as laminitis, navicular bone disease, arthropathies, pelvic limb myopathy, and back disease [14–19]. More recently, small animal veterinary studies have used MITI to evaluate a variety of conditions such as canine elbow dysplasia, intervertebral disc disease, cranial cruciate disease and feline hyperthyroidism [20–24]. Thermography utilizes Planck’s law which states that all objects above absolute temperature (0° Kelvin) emit infrared radiation [25]. A thermographic camera detects thermal radiation and produces a color map called a thermogram. Injury or disease can affect the sympathetic autonomic nervous system, which directly controls local dermal microcirculation and alters surface body heat [18, 26]. Regions of elevated temperature are indicative of increased local dermal circulation or metabolic rates secondary to disease processes such as inflammation while regions of decreased temperature are associated with reduced cutaneous perfusion and are suggestive of vascular occlusion or imbalance in parasympathetic and sympathetic input to peripheral vasculature [15, 18, 27]. Normal patients demonstrate a high degree of thermal symmetry between ipsilateral and contralateral sides [28–31]. Asymmetrical thermal patterns are suggestive of a disease process [26, 29, 31].

The purpose of this prospective study was to determine the viability of using MITI to identify changes in thermographic patterns as well as average temperature differences in a region of interest (ROI) affected by canine appendicular bone neoplasia. We hypothesized that the presence of bone cancer is associated with a change in thermographic pattern as well as an increase in surface temperature of the ROI.

## Results

### Demographics

Forty dogs were included in this study. The breeds included were: Golden Retriever (*n* = 7; 17.5%), mixed breed (6, 15%), Labrador Retriever (5; 12.5%), Rottweiler (5, 12.5%), Mastiff (4, 10%), Bulldog (2; 5%), Greyhound (2; 5%), Leonberger (1; 2.5%), Presa Canario (1; 2.5%), St. Bernard (1; 2.5%), German Shepherd (1; 2.5%), Jack Russell Terrier (1; 2.5%), Pit bull (1; 2.5%), Cocker spaniel (1; 2.5%), Border Collie (1; 2.5%), and West Highland White Terrier (1; 2.5%). Eighteen (45%) were neutered males, 17 (42.5%) were spayed females, five (12.5%) were intact males, and one (2.5%) was intact female. The average age was 8.6 years old (SD = 2.8 years). The most common bone tumors were osteosarcoma (*n* = 29, 72.5%) and spindle cell sarcoma (*n* = 4, 10%). Seventeen (42.5%) were located on the right thoracic limb, eight (20%) were located on the left thoracic limb, eight (20%) on the right pelvic limb, seven (17.5%) were located on the left pelvic limb. Based on physical exam and radiographs, no patients had bilateral appendicular bone cancer nor contralateral abnormalities of any origin.

### Color normalization and best view

**Fig. 1:**
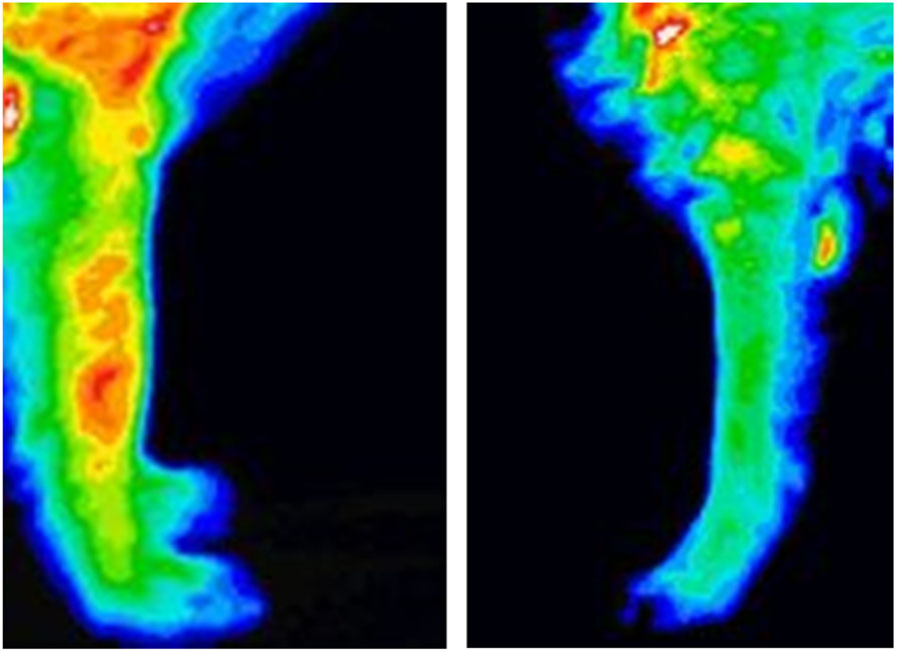
Thermographic images of the forelimb. The left image is representative of a patient with osteosarcoma of the right radius. The right image is representative of a normal contralateral left antebrachium

Eighty different experiments were used to determine the best color normalization and best thermographic view to identify a neoplastic lesion. Certain views such as the medial view or the cranial and caudal view of the proximal aspect of the limb were vastly underpowered and excluded from the experiments. Five different color normalization methods were used. NormRGB yielded the best result when used to evaluate the middle aspect of the limb (elbow/stifle) while normRGB-lum was best to evaluate the proximal (shoulder/hip), distal (carpus/tarsus), and entire limb (Fig. 1). The best imaging view that maximized thermographic differences was dependent on the body part of interest. When evaluating the entire limb, pattern analysis software had 90% success (100% sensitivity; 80% specificity) at differentiating thermographic pattern differences between cancerous and normal limb. The cranial view provided the best results when the pattern analysis software evaluated the middle aspect of the limb (elbow/stifle) and the entire limb. The lateral view provided the best results when evaluating the proximal aspect of the limb (shoulder/hip). The caudal view provided the best results when evaluating the distal aspect of the limb (carpus/tarsus) (Table 1).

**Table 1:** Color normalization and image views that yielded best experimental success rate in differentiating thermograms of canine limbs affected with bone neoplasia from that of their normal contralateral limbs

Aspect of the limb	Best view	Color normalization	Overall success	Sensitivity	Specificity
Proximal (shoulder/hip)	Lateral	normRGB-lum	75%	70%	80%
Middle (elbow/stifle)	Cranial	normRGB	88%	80%	95%
Distal (carpus/tarsus)	Caudal	normRGB-lum	100%	100%	100%
Full limb	Cranial	normRGB-lum	90%	100%	80%

### Thermographic differences between cancer and normal contralateral side

Thermographic images were evaluated based on their minimum, maximum, and average temperature of a ROI. The laterality (left or right limb) was included in the original model but was not found to be significant and was therefore, excluded in the final models. There were no significant differences when comparing the minimum temperature of the cancerous to the non-cancerous side (*p* = 0.657) or when comparing the maximum temperature of the cancer to the non-cancerous side (*p* = 0.1241). However, there was significant difference (*p* = 0.0005) when comparing the average temperature of the ROI of the cancerous to non-cancerous side (Table 2). The average surface temperature of the ROI affected by bone neoplasia was 0.53 °C (standard error = 0.14) higher than its respective normal contralateral area.

**Table 2:** Surface body temperature data of canine limbs affected with bone neoplasia and their normal contralateral limb

Temperature	Cancer ^a^	Contralateral	Paired Difference ^b^	*P*-value
Minimum (°C)	24.83 ± 0.37	24.76 ± 0.37	0.07 ± 0.16	0.6571
Maximum (°C)	31.53 ± 0.39	31.23 ± 0.39	0.37 ± 0.24	0.1241
Average (°C)	28.56 ± 0.43	28.01 ± 0.43	0.53 ± 0.14	0.0005

^a^ Mean and standard error based on nested MMANOVA models

^b^ The difference between cancer and non-cancer means may not be identical to the difference computed by pair matched analysis due to rounding error

## Discussion

This study revealed that pattern recognition software can be used to successfully differentiate between thermographic patterns of a limb affected by bone neoplasia and its normal contralateral limb. With an overall success rate ranging from 75 to 100%, this study documents that the presence of appendicular bone neoplasia is associated with a change in thermographic pattern. The best results were dependent on the various factors such as the location of the lesion (proximal, middle, distal, full), view obtained (cranial, caudal, medial, lateral), and color normalization (normRGB, normRGB-lum) (Tables 1, 3). Caudal views of the distal limb (carpus) yielded the highest sensitivity (100%) and specificity (100%) for detection of bone cancer in that area but may be the result of having the smallest sample size. Overall, it was noted that the success rate decreases as the region of interest became more proximal. This may be due to the proximity of other body parts, such as the thorax or abdomen, which may have interfered with the thermographic pattern and thus altered the pattern analysis.

**Table 3:** Number of thermographic ROI from 40 dogs affected with bone neoplasia and their normal contralateral limb

Aspect of the limb	Limb	Cranial	Lateral	Medial ^a^	Caudal	Total
Proximal (shoulder/hip)	Cancer	3 ^a^	20	2 ^a^	3 ^a^	28
	Contralateral	2 ^a^	20	2 ^a^	1 ^a^	25
Middle (elbow/stifle)	Cancer	20	29	5 ^a^	17	71
	Contralateral	21	31	4 ^a^	17	73
Distal (carpus/tarsus)	Cancer	12	15	3 ^a^	7	37
	Contralateral	11	14	3 ^a^	8	36
Full limb	Cancer	5	16	2 ^a^	2	25
	Contralateral	5	17	2 ^a^	2	26
Total	Cancer	40	80 ^b^	12 ^a^	29	161
	Contralateral	39	82 ^b^	11 ^a^	28	160

^a^ Due to small data sets, these values were not included in the analysis

^b^ Certain neoplastic lesions intersected multiple region of interests (e.g. proximal and middle limb thermographic images were taken for the same mid-diaphyseal humeral neoplasm)

Another salient finding was that the ROI average temperature (paired difference: 0.53 °C; standard error: 0.14) was significantly different between the affected ROI and normal contralateral ROI (*P* = 0.0005). Previous canine thermographic studies have not found consistent results regarding changes in the average surface temperature of the ROI. Only one study comparing the difference between canine dysplastic elbows and normal elbows noted a significant temperature change between the two groups [21]. Another study that evaluated thermographic changes between normal canine stifles and those with cranial cruciate ligament rupture found no statistical difference between the two groups [24]. This discrepancy may be the result of anatomic differences in the autonomic regulation of the various aspects of the appendicular skeleton or because of differences in how the various disease processes may impact the autonomic nervous system. In this study, the presence of bone neoplasia was associated with an increase of the average temperature by 0.5 °C in the region of interest. This finding can be advantageous for a clinician that desires a quick preliminary analysis or does not have access to pattern recognition software. Like the thermographic pattern changes associated with bone cancer, this variation in average temperature was nonspecific to bone cancer and could be a result of other pathological reasons.

Both thermographic findings revealed that MITI was sensitive enough to detect surface body temperature changes secondary to bone cancer. However, no further analyses were performed to determine differences between the various bone neoplasms. Of the forty patients in this study, 29 were affected by osteosarcoma while the remaining 11 patients were affected by 7 different cancers. Due to this large discrepancy of patients within each neoplastic subset, it was determined that further analyses would be of limited value.

It has previously been documented that clipping fur from a ROI could raise the absolute surface temperature but did not impact the relative change in surface temperature or thermographic pattern when comparing a diseased limb to its normal contralateral limb [24, 30]. As a result, this study did not clip the fur over the ROI prior to obtaining thermographic data. However, it is a possibility that fur length and density may interfere with determining surface temperature and thermographic patterns.

Historically, MITI had been used in veterinary and human medicine to identify changes associated with pathology prior to clinical signs [32, 33]. Early uses of thermography in cases of human osteosarcoma were able to detect evidence of metastasis or recurrence before other modalities could [32]. Since then, other imaging tools, such as nuclear scintigraphy, CT, MRI, PET, and SPECT, have been used to detect bone neoplasia in humans [34]. In human medicine, MITI can still be used as a screening tool for neoplasia such a breast cancer [35].

The findings of this study support the utility of thermography as a diagnostic screening tool for canine bone cancer. Despite its nonspecific nature, MITI can be a beneficial tool to use in most clinical settings. MITI is an inexpensive test that can be affordable to a wider population of owners and clinicians. Compared to the other modalities, MITI does not require sedation and will spare patients from radiation as well as the support staff from inadvertent exposure. Most importantly, it can be used for patients that present with a non-localizable source of lameness or for early screening for appendicular bone neoplasms. Serial MITI can be considered to monitor disease progression for patients undergoing palliative care. However, it must be emphasized that other imaging modalities (e.g. radiography) and procedures (e.g. bone biopsy) are required to accurately diagnose appendicular bone neoplasia. Hence, thermography is best used in conjunction with other diagnostic tools for a comprehensive diagnostic work-up.

The limitations of this study include the patient population as well as the thermographic views for certain regions of the body.

To the authors’ knowledge, there were no previous veterinary thermographic studies with respect to bone neoplasia. In order to reduce the number of variables for this study, only patients with unilateral disease and without evidence of local or distant metastasis were included. As a result, it is not documented how local or distant metastasis may affect thermograms. While it is expected for those thermograms to appear different compared to that of an unaffected limb, a study that included patients with metastasis would be needed to quantify the difference.

Another limitation with the patient population was the overwhelming number of patients affected by osteosarcoma compared to the small number of patients afflicted by another neoplasms. This prevented further subset analysis of the surface temperatures and thermographic patterns of different cancers. For a study that may want to evaluate these differences, a significantly larger patient population will be required so that the individual neoplastic subsets would not be underrepresented.

This study also did not include patients with benign causes of lameness such as an inflammatory or infectious process. As a result, it is unknown how the thermograms of those patients may compare to that of those with bone cancer.

Accurate multiplanar images were more difficult to obtain in certain regions of the body. Cranial and caudal views of the hip and shoulder included infrared data from adjacent body parts such as the head, body, or tail. As a result, these areas were limited to thermographic images taken from the lateral aspect. Images taken from the medial aspect also encountered a similar situation with thermal data from the chest or abdomen as well as interference from the presence of the contralateral limb. Future studies can consider using other views such as oblique craniolateral or caudolateral views or using light sedation to allow for the positioning of the limbs to minimize interference from other body parts.

## Conclusions

It was concluded that the presence of bone neoplasia in the canine appendicular skeleton could affect the average thermal surface temperature as well as the thermographic pattern of the limb. MITI may be used as a screening tool for primary canine appendicular bone tumors. Further studies should consider correlation between serial thermography and progression of disease. Data could potentially be extrapolated to determine a time frame to screen for appendicular bone neoplasia in non-clinical patients or those that present with subtle lameness. Another study to consider would be to determine differences in thermographic patterns associated with an appendicular bone neoplasia compared to other injuries or disease processes.

## Methods

### Sample size

The sample size of this study was based on similar veterinary studies utilizing MITI [21–24].

### Inclusion criteria

From February 2011–August 2013, 40 consecutive client-owned dogs with suspected primary appendicular bone tumors were evaluated at Long Island Veterinary Specialists by a board-certified surgeon. An index of suspicion for bone cancer was based on signalment, history, clinical signs, and radiographs with appendicular osteolytic lesions. Patients were included in the study if they were unilaterally affected, did not have enlarged peripheral lymph nodes or radiographic evidence of pulmonary metastasis, and did not have lameness or radiographic abnormalities (e.g. osteoarthritis, soft tissue lesions, etc.) of the contralateral limb. All dogs were confirmed to have bone neoplasia based on histopathologic evaluations of submitted biopsy samples. *Infrared thermography imaging.*

Portable digital infrared thermal imaging (DITI) system (Med2000 IRIS, Meditherm, Inc., Beaufort, NC) with a focal plane array amorphous silicone microbolometer was used. This system has accuracy of 0.01 °C and can measure temperature between 10°-55 °C.

**Fig. 2:**
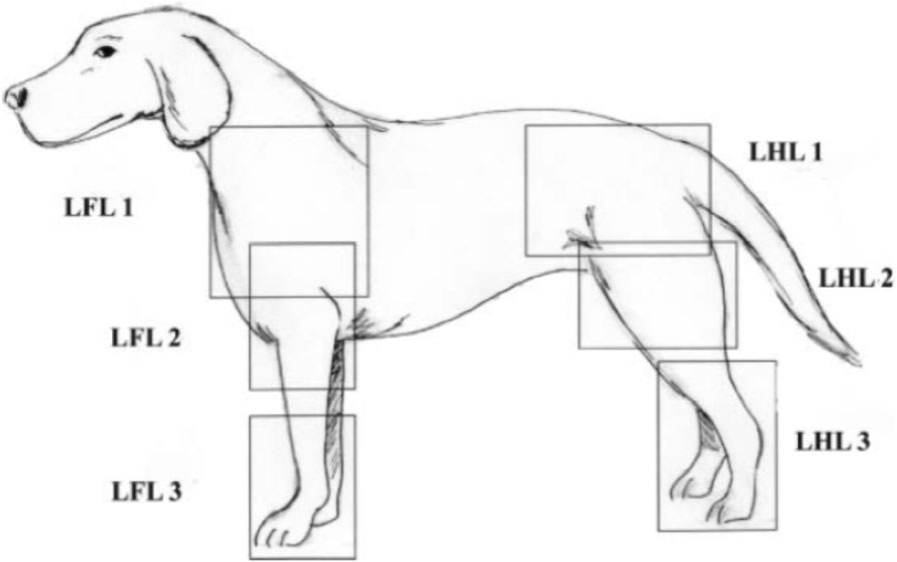
Thermographic regions of interests (ROI) of the left forelimb (LFL) and left hindlimb (LHL). The proximal, middle, and distal ROI are labeled as LFL1/LHL1, LFL2/LHL2, and LFL3/ LHL3, respectively. Illustrated by CL

All thermograms were obtained prior to shaving the area of interest and obtaining a bone biopsy. All dogs had limited exercise and thermal images were captured in the same room with ambient temperature set to 21 °C. In order to mitigate thermal artifacts from physical contact, trained technicians wore latex gloves at all times and handled dogs using leashes and by their tail. All images were taken with the patient in a standing position. Camera position was typically 1.5–4.6 m away from the patient depending on camera view. Depending on the location of the tumor, most ROI’s were isolated to either the proximal (LFL1/LHL1), middle (LFL2/LHL2), and/or distal (LFL3/LFL3) aspects of the limb (Fig. 2). In certain cases, the ROI pertained to the entire limb. If possible, multiplanar (cranial, caudal, medial, and lateral) views were taken of each specific ROI (Fig. 2). Since some lesions were located in areas that intersected two ROI’s, multiple thermographic images may have been taken of these lesions (e.g. LFL1 and LFL2 images were taken for a mid-humeral lesion). A total of 161 usable thermographic images of the affected limb and 160 usable thermographic images of the normal contralateral limb were captured (Table 3).

The thermographic camera was connected to a laptop computer for real-time data analysis. Each thermogram generated TIFF images, which were saved within the software program (WINTES 2, Compix Inc., Lake Oswego, OR) for further review and analysis. Images were classified based on location (proximal- shoulder/pelvis; middle- elbow/stifle; distal- carpus/tarsus; full limb-forelimb/hindlimb) and view (cranial, caudal, medial, lateral) (Fig. 2). Images were preset to 8 °C with a 16-shade color map. White and red represented warmer temperature and black and blue represented cooler ones. The maximum, minimum, and average temperature of the ROI was noted for each image taken. Custom image recognition software (CVIPtools, Computer Vision and Image Processing Laboratory, Department of Electrical and Computer Engineering, School of Engineering, Southern Illinois University, Edwardsville, IL) was used for quantitative analysis and pattern recognition of the thermograms.

### Imaging analysis

CVIPtools, Computer Vision and Image Processing Feature Extraction and Pattern Classification (CVIP-FEPC) and Color Normalization tools were used for thermographic analysis. The thermograms were remapped based on the minimum and maximum temperature of the experimental set. Five different color normalization methods were tested (no normalization; luminance; normGrey; normRGB; normRGB-lum). Thermograms may have different colors representing the same thermal value, depending on the settings used. Color normalization factored these differences and adjusted the images to maximize visual information (Fig. 1). Five sets of experiments, with each set being preprocessed with a different color (temperature) normalization method, were performed with the CVIP-FEPC, with each set of original images having 1023 permutations, and color normalized images having 2046 permutations. Nearest Neighbor (NN) and K-Nearest Neighbor (KNN), with K = 5, were used as the classification methods. Optimal image selection and testing were performed with the ‘leave-one-out’ cross-validation method.

### Statistical methods

The paired difference between the cancer and non-cancer side was calculated for each ROI. A positive paired difference corresponded to the cancer side being hotter than the healthy side while a negative paired difference corresponded to the healthy side being hotter than the cancer side.

Since a given patient could have multiple sites being measured (i.e. these 40 dogs contributed a total of 158 sites), a nested analysis of variance with a mixed model approach (MMANOVA) was performed to account for the correlated data within a subject. Minimum, maximum, and average temperatures of these ROI’s were analyzed.

A result was considered statistically significant at the *P* < 0.05 level of significance. All analyses were performed using SAS version 9.3 (SAS Institute Inc., Cary, NC).

## Data Availability

The datasets used and/or analyzed during the current study are available from the corresponding author upon reasonable request.

## Abbreviations

CT: Computed tomography
DITI: Digital infrared thermal imaging
IRT: Infrared thermography
MII: Medical infrared imaging
MITI: Medical infrared thermal imaging
MRI: Magnetic resonance imaging
PET: Positron emission tomography
ROI: Region of interest
SPECT: Single-photon emission computed tomography
